# Leg Dominance and OSB12 Kick Start Performance in Young Competitive Swimmers

**DOI:** 10.3390/ijerph182413156

**Published:** 2021-12-14

**Authors:** Ivan Matúš, Pavel Ružbarský, Bibiana Vadašová, Pavol Čech

**Affiliations:** Faculty of Sports, University of Presov, 08001 Presov, Slovakia; pavel.ruzbarsky@unipo.sk (P.R.); bibiana.vadasova@unipo.sk (B.V.); pavol.cech@unipo.sk (P.Č.)

**Keywords:** biomechanics, kinematic analysis, start phase, leg positioning

## Abstract

The purpose of this study was to examine differences in starting and kick-plate positions, pointing to an effect on kick-start performance with the dominant and non-dominant feet placed on the front edge of the OSB12 starting block. The sample included 20 female competitive swimmers whose average age was 16.1 ± 0.6 years. To assess swimmers’ lower body explosive strength and determine leg dominance, a triple hop distance test was administered. We determined the swimmers’ preferred starting position on a starting block in terms of the kick-plate setting and body position on the starting block. The results of our study demonstrate the importance of leg dominance and positioning on the OSB12 starting block. After the preferred starting position was determined, the optimal position for the kick start was selected. When the dominant leg was placed on the front edge of the starting block (*p* < 0.05; Cohen’s *d*—large effect), swimmers produced shorter block times and times to 2 and 5 m. For most of the tested swimmers, the optimal basic position on the starting block included neutral- and rear-weighted positions with the kick plate set to positions 3–4 and the right leg placed on the front edge of the OSB12 starting block.

## 1. Introduction

The new starting platform OSB11 by Omega is currently used in national and international swimming competitions. The unique feature of the Omega^®^ OSB11 is the kick plate angled at 30° from the platform surface [1]. The kick plate can be shifted front to back to five different locations that span 0.2 m, and the platform is angled at 9° from the horizontal [2]. Most recent studies have shown that the addition of a kick plate to the block may allow swimmers to more effectively apply force during the start. Improved start performance was determined in particular by a shorter block time and higher take-off velocity [1,3,4,5,6,7,8,9,10,11]. When comparing start techniques such as track start, grab start, and kick start, multiple authors found shorter times to 2, 5, 7.5, 10, and 15 m when the swimmers used the kick start. Therefore, swimmers should learn to perform the correct kick-start technique [1,3,4,9,11,12]. Swimmers need to realize that a change in the foot placement on the kick plate may change their swim start performance [13]. From the viewpoint of lower leg dominance and leg positioning on the starting block, Hardt et al. [14] found that the dominant track start stance, be it left foot forward or right foot forward, was not related to any of the footedness measures when starting from the previous model of the starting block. A study by Slawson et al. [13], where swimmers started from the OSB11 starting platform, showed that head distance of entry was greater on average for dives in which swimmers had dived with their right leg forward as opposed to their left. Leg dominance was not determined, but there were differences in the start performance depending on which leg was placed on the front edge of the starting block.

Most studies differ in the starting block used, and only replicas of the OSB12 starting block were used in multiple studies [9,10,13,15,16]. From this viewpoint, various studies, where the replicas of OSB11 and OSB12 starting blocks were used, have shown different results for kick-start performance. Despite the interesting findings reported, all studies dealt with three kick plate positions out of three (3 to 5). In some studies, the kick plate was set to ±1 from the preferred position. Therefore, these studies do not provide a complex overview of various leg angles and kick-plate positions during the takeoff from OSB12. When determining the start position (front-, neutral-, and rear-weighted) on the OSB11 starting block, no studies [17,18,19] dealt with changes in the center of gravity for all kick-plate positions on the OSB11 and OSB12 starting blocks. The determination of the starting position from OSB11–12 was different as well. Coaches and swimmers choose the leg positioning based on subjective preferences rather than objective data.

The purpose of this study was to examine differences in starting and kick-plate positions, pointing to an effect on kick-start performance with the dominant and non-dominant feet placed on the front edge of the OSB12 starting block.

## 2. Materials and Methods

### 2.1. Participants

The sample included 20 female competitive swimmers (whose average age, height, and weight were *x* = 16.1 ± 0.6 years, *x* = 170.2 ± 3.4 cm, and *x* = 57.1 ± 2.7 kg, respectively). The swimmers participated regularly in the Slovak regional swimming championships and the Slovak swimming championship, having competed specifically in sprint races and freestyle races (50 m free style 27.92 ± 0.89 s). All swimmers were healthy and did not report any health problems before testing.

Ethical approval for this study was obtained from the Ethics Committee of the University of Presov, Slovakia (approval no.: 1/2021). All swimmers who participated in this study read an information leaflet about testing and measurement and provided their informed written consent. Participation in the study was fully voluntary and anonymous, with no explicit incentives provided for participation.

### 2.2. Test Protocol

#### 2.2.1. Lower-Body Explosive Strength Test

To assess the swimmers’ lower-body explosive strength and determine leg dominance, the triple hop distance test was administered. This test has been shown to be a reliable test of lower leg strength and power [20]. We fixed a tape measure to the ground, perpendicular to a starting line. Swimmers stood on the designated testing leg, with the large toe on the starting line, and performed three consecutive maximal hops forward on the same (dominant) leg. Arm swing was allowed. The investigator measured the distance hopped from the starting line to the point where the heel struck the ground upon completing the third hop. All swimmers were allowed one to three practice trials on each leg and then completed three test trials [21]. The test was administered one day before testing in the water.

#### 2.2.2. Kick-Start Trials

Before performing kick starts, swimmers warmed up on dry land for 15 min and then swam 400 m. After the warmup, 11 waterproof adhesive markers were applied on the swimmers’ bodies [22] in the following locations—lateral margin of the left transverse tarsal joint, lateral left and right malleolus, lateral left and right knee condyle, left and right greater trochanter, lateral margin of the left and right scapular spine, lateral left and right elbow epicondyle, ulnar styloid process of the left and right wrist, and medial side of the fifth metacarpal–phalanx joint. Then, the swimmers performed three trial kick starts from the OSB12 starting block to become familiar with the three basic starting positions: front-, neutral-, and rear-weighted.

To determine the starting position, we placed a 2 cm-thick bar perpendicular to the starting block’s front edge. The body position in the starting block’s basic position was determined according to the spot marked on the scapular spine as front- (located in front of the bar), neutral- (overlapped with the bar), and rear-weighted (located behind the bar). Swimmers took their marks and responded to a sound signal and an LED light signal at the same time. The swimmers started from starting positions and set the kick plate to positions 1–5. Each swimmer performed three starts from all three positions (front-, neutral-, and rear-weighted). One kick-start trial required 1.7 s (block phase—0.7 s; flight phase—0.4 s; underwater phase—0.5 s). The swimmers expended energy by the performing movement on the starting block, which took 0.4 to 0.5 s. Swimmers were instructed not to perform any kicking or undulating movements during the underwater phase. The rest period between starts and changes in the OSB12 kick-plate position was 30 s and 2 min, respectively. The rest period after nine kick-start trials was 5 min. The swimmers followed the same order to ensure recovery. Each swimmer performed 45 kick starts evenly distributed over two days at the same time of day. Swimmers were divided into two similar groups. The first group performed the kick-start trial before midday and the second group in the afternoon. The order of start positions and kick-plate positions was randomized.

The next day, after finding the start position with the shortest time to 5 m determined for all swimmers, we aimed to determine which leg (dominant or non-dominant) should be placed on the front edge of the starting block. Swimmers assumed a starting position on the starting block by exchanging the position of their legs in the starting position. The leg placed on the front edge of the OSB12 was placed against the kick plate and vice versa. In these positions, swimmers performed three kick starts with the same duration of kick start and rest periods as when the optimal starting position was determined.

The kick-start parameters assessed in this study included front knee angle (FKA), front ankle angle (FAA), rear knee angle (RKA), rear ankle angle (RAA), hip angle (HA), block time (BT), take-off angle (TA), head position (HP), times to 2 m and 5 m (T2 and T5), entry angle (EA), flight time (FT) and distance (FD), glide time (GT), glide distance (GD), and maximal depth (MaxH).

We used the SwimPro five-camera system (50 fps; shutter speed 1/1000 s) to measure the kick-start parameters. The first camera was perpendicular to the starting block at a 0 m distance from the pool’s edge and 1.5 m above the water surface. The second camera was 1.6 m from the pool’s edge and 1.5 m above the water surface. The third camera was 1.6 m from the pool’s edge and 1.7 m below the water surface. The fourth camera was 5 m away from the pool’s edge and 1.7 m below the water surface. To increase the level of lighting, we used halogen and additional LED lights. Using the Dartfish© software (Dartfish ProSuite 4.0, 2005; Fribourg, Switzerland), we assessed the 2D analysis video recordings. This software meets the validity and reliability criteria for assessing kinematic parameters using 2D analysis in swimming [23,24].

### 2.3. Statistical Analysis

To determine significant differences in the selected kinematic kick-start parameters relative to the starting position, the two-sample *t*-test with equal variance was used. Significant differences were assessed at *p* < 0.05. Cohen’s *d* [25] was used to calculate the effect size (small effect = 0.2, medium effect = 0.5, and large effect = 0.8) based on the standardized difference between two means. The collected data were statistically processed using the statistical software Stata 17.

## 3. Results

The triple hop distance test scores for the right and left leg enabled us to determine dominance and showed that the right leg was dominant in 19 of 20 swimmers. Regarding the starting position on the starting block, we determined that 15 of 20 swimmers set the kick plate to position 3. Most of the swimmers preferred a neutral-weighted kick start with their right leg on the front edge of the starting block.

The measurements during kick starts with different OSB12 kick-plate positions showed that the optimal kick-start positions for the swimmers included the neutral- and rear-weighted positions with the kick plate in positions 3–4. When the kick starts were performed with either the dominant or non-dominant leg on the front edge of the starting block, the measurements showed that the dominant leg should be placed on the front edge of the starting block (Table 1).

### 3.1. Block Phase

**Figure 1:**
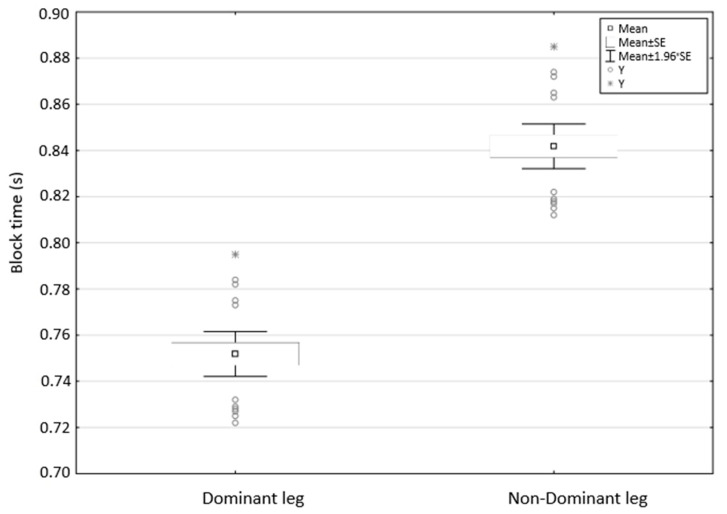
Dominant and non-dominant leg placed on the front edge-block time.

The biomechanical analysis data showed that the ankle, knee, and hip angles were not significantly different during the block phase. This demonstrated that reversal of leg positioning (dominant and non-dominant) on the front edge of the starting block did not affect the starting positions (Table 2). Regarding time, leg reversal had a significant effect (*p* < 0.05) on the block time (*p* = 0.00, *d* = −4.07—large effect). When the dominant leg was placed on the front edge of the starting block, swimmers produced shorter times (Figure 1).

### 3.2. Flight Phase

**Figure 2:**
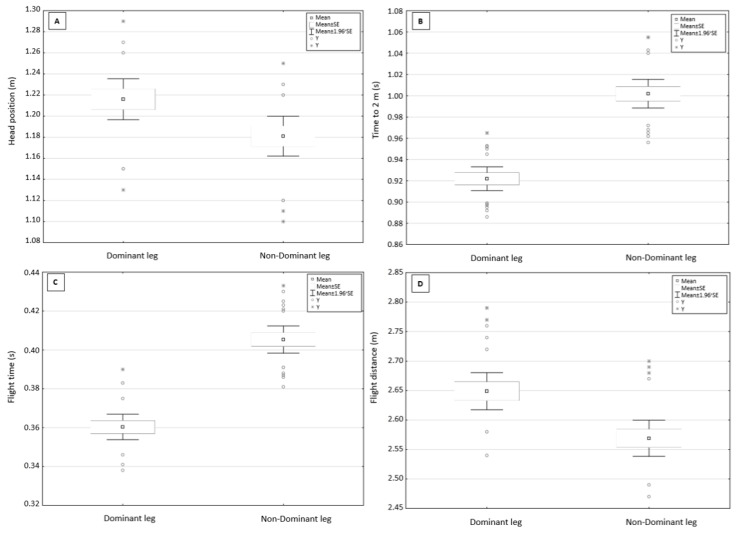
Dominant and non-dominant leg placed on the front edge—(**A**)—head position; (**B**)—time to 2 m; (**C**)—flight time; (**D**)—flight distance.

For the flight phase during the kick start from OSB11, reversal of leg positioning did not significantly affect the takeoff and entry angles. There were statistically significant differences (*p* < 0.05) in head position (*p* = 0.01, *d* = 0.80—large effect), time to 2 m (*p* = 0.00, *d* = −2.83—large effect), flight time (*p* = 0.00, *d* = −2.91—large effect), and flight distance (*p* = 0.01, *d* = 1.13—large effect; Table 3). A higher head position, shorter time to 2 m, shorter flight time, and longer flight distance were recorded when the dominant leg was placed on the front edge of the OSB11 starting block (Figure 2). 

### 3.3. Underwater Phase

**Figure 3:**
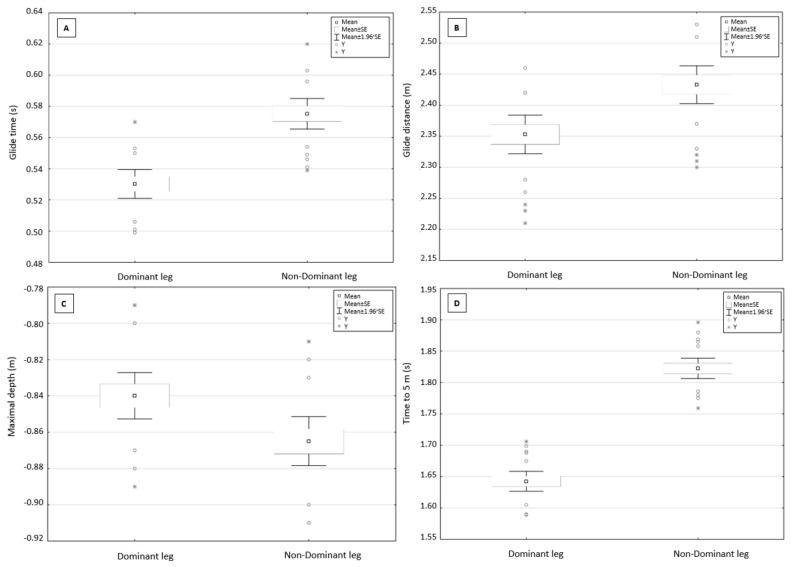
Dominant and non-dominant leg placed on the front edge—(**A**)—glide time; (**B**)—glide distance; (**C**)—maximal depth; (**D**)—time to 5 m.

For the underwater phase during kick start from the OSB11, reversal of leg positioning significantly affected glide time, glide distance, and maximum depth and time to 5 m. There were statistically significant differences (*p* < 0.05) in glide time (*p* = 0.00, *d* = −2.08—large effect), glide distance (*p* = 0.01, *d* = −1.14—large effect), maximal depth (*p* = 0.01, *d* = 0.84—large effect), and time to 5 m (*p* = 0.00, *d* = −4.92—large effect; Table 4). When the dominant leg was placed on the front edge of the starting block, swimmers produced shorter glide time and glide distance, less maximal depth, and shorter time to 5 m (Figure 3).

## 4. Discussion

This study aims to determine how leg dominance and reversal of leg positioning affect the kick-start performance from OSB12 in terms of changes in angular and kinematic parameters.

Using the triple hop distance test, leg dominance was determined. According to Hamilton et al. [21], Jones at al. [26], and Munro [27] this test is a reliable test of leg strength and power.

Before measuring kick-start parameters, we determined the swimmers’ preferred starting position on an OSB11 starting block. According to the triple hop distance test scores, three swimmers had to assume a different starting position from their preferred positions during kick start. Most of the swimmers placed their right (dominant) legs on the front edge of the starting block. Similar results were reported by Slawson et al. [13], who determined that the most notable influence of the kick-start setup was the front leg, which resulted in significant relationships for all output and performance variables tested. Barlow et al. [18] determined the regular starting position among their participants and found that six of 10 swimmers regularly used their left foot as the rear foot during a kick start. However, neither Slawson’s nor Barlow’s study aimed to determine whether the right leg placed on the front edge of the starting block was dominant or non-dominant.

Leg dominance and preferred foot position has also been explored in the track-start stance using an older model and new OSB11 start platforms. Previous research suggested that for the track start, the main task of the front leg is to generate better takeoff, which may be best achieved using the stronger leg at the front edge of the start block. The front leg has more time on the start block to produce force [3,28]. Hardt et al. [14] did not find a significant difference in start times with either foot at the front of the block when they used a previous platform model. A study by Slawson et al. [13] using the OSB11 platform demonstrated that the swimmers had greater flight distance when the right leg was placed at the front edge. This study differs from the study by Hardt et al. [14] in the tests used. Hardt et al. [14] administered the Waterloo Footedness Questionnaire—Revised and a one-legged countermovement jump to assess leg dominance and strength. For this study, we decided to administer the triple hop distance test because it is easy to administer by swimming coaches. We also found that the dominant leg should be placed on the front edge of the starting block (*p* < 0.05). Leg dominance must be based on the strength test, because the left leg can be placed on the front edge of the starting block as well.

The results of the studies by Slawson et al. [13], Honda et al. [17], and Takeda et al. [16] aimed to determine the optimal kick-plate position for more effective takeoff from the starting block. Despite their interesting results, all three studies addressed only three levels of the kick plate out of the possible five. Therefore, the studies do not offer a complete overview of the effectiveness of the various angles and levels of the kick plate when taking off from the OSB12 starting block. In this study, we performed testing to determine the optimal starting position on the starting block, which consisted of passing through the individual levels of the OSB12 kick plate for all positions (front-, neutral-, and rear-weighted). The final times showed that the optimal kick-plate position for swimmers was 3–4 for the neutral- or rear-weighted starting positions, if the dominant leg was on the front edge of the starting block and not on the OSB12 kick plate. For some swimmers, the optimal position of the kick plate was different than their preferred one, sometimes by up to two positions. The same applied to the basic starting position, because for some swimmers the optimal basic position was the rear-weighted one, with the front-weighted as the preferred one.

Other studies [13,15,16,17,18] also showed that swimmers should optimize their starting positions by setting the kick plate to positions 3–4. Swimmers produced higher horizontal take-off velocity when their center of gravity was at the back of the starting block.

We also investigated leg dominance on the front edge of the starting block during the particular phases of kick start. The results of our kinematic analysis for the block phase did not reveal any statistically significant differences between the angles of the legs. The starting position on the starting block was not affected by the dominant or non-dominant leg placement on the front edge of the starting block. We recorded a statistically significant difference (*p* < 0.05) in the block time, which was shorter when the dominant leg was in front. We found similar results for the flight phase duration, and statistically significant differences (*p* < 0.05) were determined for the maximum head position at take-off, time to 2 m, flight time, and distance. For the underwater phase, we recorded a statistically significant difference (*p* < 0.05) for the glide time and distance, maximal depth, and time to 5 m when a shorter time to 5 m was measured when the dominant leg was placed on the front edge of the OSB12 starting block. Cohen’s effect size showed a large effect on these differences. These results support the fact that the dominant leg should be placed on the front edge of the OSB12 starting block. However, it should be noted that force is also produced by the non-dominant leg, which is placed on the OSB12 kick plate. Both legs are crucial for swim start performances. A study by Slawson et al. [29] supported this fact because they found that the force produced by the rear leg placed on the OSB12 kick plate affects the starting performance. Thus, the dominant leg should be placed on the front edge of the starting block. This is associated with the end of the block phase, during which swimmers take off from the starting block and achieve the greatest acceleration [8,9,30]. This acceleration must also be controlled to make the take-off as effective as possible. According to Burkhardt et al. [31], the acute reversal of the leg positioning impairs the start performance. In this study, however, the plate-specific kinetic analysis revealed a higher horizontal peak force and impulse for the kick plate independently from the leg positioning: preferential vs. non-preferential leg positioning and strong front vs. strong back.

## 5. Conclusions

The results of our study demonstrate the importance of leg positioning on the OSB12 starting block. When the dominant leg was placed on the front edge of the starting block, swimmers produced shorter (*p* < 0.05) block times for times to 2 and 5 m. Thus, when leg dominance needs to be determined, a leg strength test must be administered by swimming coaches. Leg strength tests require minimal equipment, space, and time. For most of our tested swimmers, the optimal basic position on the starting block included the neutral- and rear-weighted positions, with the kick plate set to positions 3–4 and the dominant leg placed on the front edge of the OSB12 starting block.

## Figures and Tables

**Table 1:** Preferred and optimal kick-start positions.

Swimmer	DL	Preferred			Optimal		
		KPP	BP	FL	KPP	BP	FL
1	Right	3	NW	Right	3	NW	Right
2	Right	3	FW	Right	3	RW	Right
3	Right	3	NW	Left	3	RW	Right
4	Left	3	NW	Left	3	RW	Left
5	Right	3	FW	Right	3	NW	Right
6	Right	2	NW	Right	4	NW	Right
7	Right	3	NW	Right	3	RW	Right
8	Right	2	NW	Right	4	NW	Right
9	Right	3	NW	Right	3	RW	Right
10	Right	3	NW	Right	3	NW	Right
11	Right	2	NW	Left	3	NW	Right
12	Right	4	NW	Right	3	RW	Right
13	Right	3	NW	Right	3	RW	Right
14	Right	3	NW	Left	3	NW	Right
15	Right	3	NW	Right	3	RW	Right
16	Right	3	NW	Right	4	RW	Right
17	Right	2	NW	Right	3	RW	Right
18	Right	3	FW	Right	3	RW	Right
19	Right	3	FW	Right	3	NW	Right
20	Right	3	FW	Right	3	RW	Right

Note: DL—dominant leg; KPP—kick-plate position; BP—body position; FL—front leg; FW—front-weighted; NW—neutral-weighted; RW—rear-weighted.

**Table 2:** Effects of leg dominance on kick-start parameters—block phase.

Variables	Leg	M	SE	SD	95% CI		*t*	Prob	Prob	Prob	ES
					Lower	Upper		(T < t)	(|T| > |t|)	(T > t)	(Cohen’s *d*)
FKA	D	131.18	0.63	2.82	129.86	132.50	0.89	0.81	0.38	0.19	0.28
	Non-D	130.39	0.62	2.79	129.08	131.70					
FAA	D	125.73	0.94	4.22	123.75	127.70	0.59	0.72	0.56	0.28	0.19
	Non-D	124.94	0.94	4.21	122.96	126.91					
RKA	D	81.35	0.49	2.21	80.31	82.38	1.13	0.87	0.27	0.13	0.36
	Non-D	80.56	0.50	2.23	79.51	81.60					
RAA	D	98.17	0.69	3.08	96.73	99.61	0.82	0.79	0.42	0.21	0.26
	Non-D	97.38	0.68	3.04	95.96	98.80					
HA	D	42.87	0.39	1.73	42.06	43.68	1.43	0.92	0.16	0.08	0.45
	Non-D	42.08	0.39	1.77	41.25	42.91					
BT	D	0.75	0.01	0.02	0.74	0.76	−12.87	0.00 *	0.00 *	1.00	−4.07
	Non-D	0.84	0.01	0.02	0.83	0.85					

Note: FKA—front knee angle; FAA—front ankle angle; RKA—rear knee angle; RAA—rear ankle angle; HA—hip angle; BT—block time; D—dominant leg; Non-D—non-dominant leg; M—mean; SE—standard error; SD—standard deviation; CI—confidence interval; Prob—probability: ES—effect size; * *p* < 0.05.

**Table 3:** Effects of leg dominance on kick-start parameters—flight phase.

Variables	Leg	M	SE	SD	95% CI		*t*	Prob	Prob	Prob	ES
					Lower	Upper		(T < t)	(|T| > |t|)	(T > t)	(Cohen’s *d*)
TA	D	38.03	0.55	2.45	36.89	39.17	0.90	0.81	0.37	0.19	0.26
	Non-D	37.33	0.54	2.46	36.18	38.48					
HP	D	1.22	0.01	0.04	1.20	1.24	2.53	0.99	0.01 *	0.01 *	0.80
	Non-D	1.18	0.01	0.04	1.16	1.20					
T2	D	0.92	0.01	0.03	0.91	0.93	−8.96	0.00 *	0.00 *	1.00	−2.83
	Non-D	1.00	0.01	0.03	0.99	1.02					
EA	D	35.20	0.33	1.46	34.51	35.88	1.50	0.93	0.14	0.07	0.47
	Non-D	34.50	0.33	1.50	33.80	35.19					
FT	D	0.36	0.01	0.02	0.35	0.37	−9.20	0.00 *	0.00 *	1.00	−2.91
	Non-D	0.41	0.01	0.02	0.40	0.41					
FD	D	2.65	0.02	0.07	2.62	2.68	3.57	1.00	0.01 *	0.01 *	1.13
	Non-D	2.57	0.02	0.07	2.54	2.60					

Note: TA—take-off angle; HP—head position; T2—time to 2 m; EA—entry angle; FT—flight time; FD—flight distance; D—dominant leg; Non-D—non-dominant leg; M—mean; SE—standard error; SD—standard deviation; CI—confidence interval; Prob—probability: ES—effect size; * *p* < 0.05.

**Table 4:** Effects of leg dominance on kick-start parameters—underwater phase.

Variables	Leg	M	SE	SD	95% CI		*t*	Prob	Prob	Prob	ES
					Lower	Upper		(T < t)	(|T| > |t|)	(T > t)	(Cohen’s *d*)
GT	D	0.53	0.01	0.02	0.52	0.54	−6.57	0.00 *	0.00 *	1.00	−2.08
	Non-D	0.58	0.01	0.02	0.57	0.59					
GD	D	2.35	0.02	0.07	2.32	2.38	−3.60	0.01 *	0.01 *	1.00	−1.14
	Non-D	2.43	0.02	0.07	2.40	2.47					
MaxH	D	−0.84	0.01	0.03	−0.85	−0.83	2.64	1.00	0.01 *	0.01 *	0.84
	Non-D	−0.87	0.01	0.03	−0.88	−0.85					
T5	D	1.64	0.01	0.04	1.63	1.66	−15.54	0.00 *	0.00 *	1.00	−4.92
	Non-D	1.82	0.01	0.04	1.81	1.84					

Note: GT—glide time; GD—glide distance; MaxD—maximal depth; T5—time to 5 m; D—dominant leg; Non-D—non-dominant leg; M—mean; SE—standard error; SD—standard deviation; CI—confidence interval; Prob—probability: ES—effect size; * *p* < 0.05.

## Data Availability

The data sets generated and analyzed for this study can be requested by correspondence authors.
